# QTc Prolongation in Patients Acutely Admitted to Hospital for Psychosis and Treated with Second Generation Antipsychotics

**DOI:** 10.1155/2013/375020

**Published:** 2013-12-31

**Authors:** Erik Johnsen, Kristina Aanesen, Sanjeevan Sriskandarajah, Rune A. Kroken, Else-Marie Løberg, Hugo A. Jørgensen

**Affiliations:** ^1^Division of Psychiatry, Haukeland University Hospital, Sandviken, Norway; ^2^Department of Clinical Medicine, Section Psychiatry, University of Bergen, Norway; ^3^Faculty of Medicine and Dentistry, University of Bergen, Norway; ^4^Department of Biological and Medical Psychology, University of Bergen, Norway

## Abstract

QTc interval prolongation is a side effect of several antipsychotic drugs, with associated risks of torsade de pointes arrhythmias and sudden cardiac death. There is an ongoing debate of whether or not electrocardiogram (ECG) assessments should be mandatory in patients starting antipsychotic drugs. To investigate QTc prolongation in a clinically relevant patient group 171 adult patients acutely admitted to an emergency ward for psychosis were consecutively recruited. ECGs were recorded at baseline and then at discharge or after 6 weeks at the latest (discharge/6 weeks), thus reflecting the acute phase treatment period. The mean QTc interval was 421.1 (30.4) ms at baseline and there was a positive association between the QTc interval and the agitation score whereas the QTc interval was inversely associated with the serum calcium level. A total of 11.6% had abnormally prolonged QTc intervals and another 14.3% had borderline prolongation. At discharge/6 weeks, the corresponding proportions were reduced to 4.2% and 5.3%, respectively. The reduction of the proportion with prolonged QTc intervals reached statistical significance (chi-square exact test: *P* = 0.046). The finding of about one-quarter of the patients with borderline or prolonged QTc intervals could indicate mandatory ECG recordings in this population. This trial is registered with ClinicalTrials.gov ID: NCT00932529.

## 1. Introduction

Prolongation of the heart rate-corrected QT (QTc) interval of the electrocardiogram (ECG) is a major concern because of the associated risk of torsade de pointes (TdP) arrythmias and sudden cardiac death [1]. QTc intervals longer than 500 milliseconds (ms) or increases of more than 60 ms of the QTc interval are established thresholds for clear concern of arrhythmia but increased risk is found also at lower levels of QTc prolongation [2]. The QT interval represents the onset of electrical depolarisation of the ventricles to the end of repolarisation of the heart [1, 2] and is influenced by both physiological and pathological factors including emotional stress, gender, obesity, food consumption, and electrolyte disturbances, as well as diseases of the heart muscle and coronary artery disease [2–4]. Moreover, several drugs including psychotropics can induce prolongation of the QTc interval principally by blocking the rapidly activating potassium current [2, 5, 6]. Antipsychotic drugs are associated with dose related QTc prolongation, and some agents have been intermittently or permanently withdrawn from the market for this reason [2, 5, 7]. Treatment recommendations for schizophrenia differ substantially, however, with regard to whether or not ECG recordings should be routinely undertaken in all patients starting antipsychotic drug treatment [8–12]. Some recommend ECG recordings in patients considered at increased risk from QTc prolongation only [8–10], whereas others recommend mandatory ECG in all patients [11], or in those admitted to hospital [12]. Importantly, patients with schizophrenia may have a heightened background risk for QTc prolongation irrespective of the drug effects, as recent studies report considerably increased prevalence of cardiovascular disease (CVD) in schizophrenia [13, 14] and that CVD is suboptimally addressed in this population [15, 16]. Active psychosis may further increase the risk of QTc prolongation because of the associated emotional stress involved although the literature is conflicting. Bär et al. [17] found increased QT variability but shorter QT mean intervals in 25 unmedicated schizophrenia patients with acute psychosis compared to healthy controls. Hatta et al. [18] on the other hand found in their cross-sectional study prolongation of the QTc interval in unmedicated acute psychotic emergency patients, all of whom were involuntarily admitted because of immediate danger to themselves or others. Patients with prior drug abuse or alcohol dependence were excluded. A major obstacle might be that most available studies include selected samples which could bias the results. Taken together acute admission to hospital may represent a particular risk situation for QTc prolongation in schizophrenia patients. To the best knowledge of the authors, studies investigating the QTc interval in samples representative of acutely admitted patients with psychosis are scarce as patients with, for example, illicit drug abuse and somatic disease are typically excluded in antipsychotic drug studies [19].

The primary aims of the study were to investigate the proportions with prolonged QTc interval at hospital admission and discharge or at 6 weeks from baseline at the latest, if not discharged earlier (discharge/6 weeks). Secondary aims were to assess the influence of electrolyte levels and emotional stress at baseline and antipsychotic drug use at discharge/6 weeks on the QTc interval. The patient recruitment focused on all patients with psychosis acutely admitted to the emergency ward to ensure a clinically relevant sample.

## 2. Materials and Methods

The materials and methods used have been described in greater detail elsewhere [20]. The study is part of a pragmatic, randomized trial comparing second generation antipsychotics (SGAs) in the treatment of psychosis including 226 patients. The present paper reports data obtained at baseline and discharge/6 weeks in patients who underwent ECG recordings. Patients were consecutively recruited from March 2004 until February 2009 from the Haukeland University Hospital with a catchment population of about 400,000. The study was approved by the Regional Committee for Medical Research Ethics and the Norwegian Social Science Data Services. The study was publicly funded and has not received any financial or other support from the pharmaceutical industry. The Regional Committee for Medical Research Ethics allowed eligible patients to be included before informed consent was provided, thus entailing a clinically relevant representation in the study. All adult patients were eligible for the study if they were acutely admitted to the emergency ward for symptoms of active psychosis as determined by a score of ≥4 on one or more of the items delusions, hallucinatory behavior, grandiosity, suspiciousness/persecution, or unusual thought content in the PANSS [21] and were candidates for oral antipsychotic drug therapy. Accordingly the patient inclusion encompassed the consecutive recruitment of a clinically representative sample of psychosis patients acutely admitted to hospital. All eligible patients met the ICD-10 diagnostic criteria (http://apps.who.int/classifications/icd10/browse/2010/en) for schizophrenia, schizoaffective disorder, acute and transient psychotic disorder, delusional disorder, drug-induced psychosis, bipolar disorder except for manic psychosis, or major depressive disorder with psychotic features. The diagnoses were determined by the hospital's psychiatrists or specialists in clinical psychology. Patients were excluded from the study if they were unable to use oral antipsychotics, were suffering from manic psychosis, or for other behavioural or mental reasons related to the state of illness were unable to cooperate with assessments, did not understand spoken Norwegian, were candidates for electroconvulsive therapy, or were medicated with clozapine on admittance. Patients with drug-induced psychoses were included only when the condition did not resolve within a few days and when antipsychotic drug therapy was indicated.

### 2.1. Assessments

Assessments were conducted at baseline and discharge/6 weeks. Before inclusion, eligible patients underwent the PANSS structured clinical interview. Intraclass correlation coefficients (ICC) were calculated based on interrater assessments and showed high interrater reliability (0.92). The PANSS excited component (PANSS-EC), consisting of the PANSS items P4 (excitement), P7 (hostility), G4 (tension), G8 (uncooperativeness), and G14 (poor impulse control) as validated by Montoya et al. [22], was used as a proxy for emotional stress. Furthermore, the Calgary Depression Scale for Schizophrenia (CDSS) [23] and the Clinical Drug and Alcohol Use Scales (CDUS/CAUS) [24] were used, as well as a neurocognitive test battery [25], and the patients were rated according to the Clinical Global Impression—Severity of Illness scale (CGI-S) [26], and the Global Assessment of Functioning—Split Version, Functions scale (GAF-F) [27]. A blood sample was collected from the patients between 08 and 10 a.m. for analyses of serum potassium, sodium and calcium, and serum level measurements of antipsychotics at discharge/6 weeks.

Drug doses were converted to mean defined daily doses (DDDs) as developed by the World Health Organization Collaborating Centre for Drug Statistics Methodology [20]. The basic definition of the DDD unit is the assumed average maintenance dose per day for a drug used for its main indication in adults.

### 2.2. QTc Assessments

The QTc interval estimation was done automatically by a Philips Pagewriter Trim II cardiograph at admission and discharge/6 weeks. Bazett's formula was used for correction. The ECG recording at baseline was done before the first administration of the study SGAs. The QTc ratings were for each gender divided into normal, borderline, and prolonged groups according to the Committee for Proprietary Medicinal Products (http://www.fda.gov/ohrms/dockets/ac/03/briefing/pubs/cpmp.pdf). The cutoff points were for men less than 430 milliseconds (ms) (normal), 430 to 450 ms (borderline), and more than 450 ms (prolonged) and for women less than 450 ms (normal), 450–470 ms (borderline), and more than 470 ms (prolonged).

### 2.3. Statistical Procedures

Categorical and continuous data at baseline and at discharge/6 weeks, respectively, were analyzed by means of exact *χ* ^2^-tests, independent samples *t*-tests, and one-way ANOVAs by using the SPSS software, version 20.0 (IBM SPSS Statistics, 2011). For comparing mean QTc intervals at baseline and at discharge/6 weeks, paired-sample *t*-tests were used. To investigate the association between QTc intervals and the levels of individual electrolytes, as well as the PANSS-EC, a bivariate analysis of correlation was performed using the Pearson correlation coefficient as normal distribution was assumed. These variables were also analysed collectively by means of linear regression. The level of statistical significance was set at *α* = 0.05, two-sided.

## 3. Results

A total of 171 patients (75.7% of the total sample) underwent ECG recordings on at least one of the time points. In the patients without ECG recordings the principal reasons were refusal or inability to cooperate with the measurements. Baseline clinical and demographic characteristics are displayed in Table 1, and there were no differences between those with and without ECG recordings. Those without ECG recordings had a numerically higher mean PANSS-EC score compared to those with ECG recordings but the difference did not reach statistical significance (independent samples *t*-test, equal variances assumed: *P* = 0.126; mean difference 0.72; 95% confidence interval (CI) −0.20–1.64).

**Figure 1:**
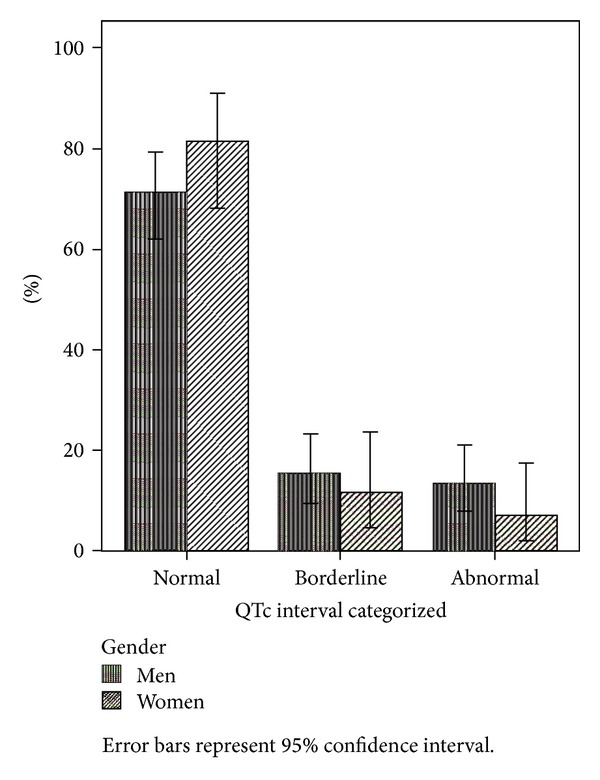
Distribution of QTc intervals at baseline.

The mean QTc interval was 421.1 (30.4) ms at baseline, and 11.6% of the patients had abnormally prolonged QTc (Figure 1). Another 14.3% had borderline QTc intervals. Two patients had QTc intervals above 500 ms at baseline.

There were no statistical differences among the genders or among the drug naïve and those with prior antipsychotic drug exposure with regard to mean QTc interval or proportion with QTc prolongation. Mean serum levels with standard deviations (SD) and reference ranges in brackets of sodium, potassium, and calcium were 141.3 (2.3) (137.0–145.0), 4.3 (0.4) (3.5–5.0), and 2.4 (0.1) (2.2–2.6) nanomoles per litre, respectively. There was a statistically significant negative association between QTc interval and serum calcium level (Pearson correlation: *r* = −0.186, *P* = 0.027), whereas no association was found between the QTc interval and sodium or potassium, respectively. There was a positive association between the PANSS-EC and the QTc interval (Pearson correlation: *r* = 0.170, *P* = 0.040). When the electrolytes and the PANSS-EC were entered as independent variables into a linear regression model with the QTc interval as the dependent variable the association between serum calcium and the QTc interval remained unaltered whereas the correlation between the PANSS-EC and the QTc interval increased (Pearson correlation: *r* = 0.205, *P* = 0.008).

**Figure 2:**
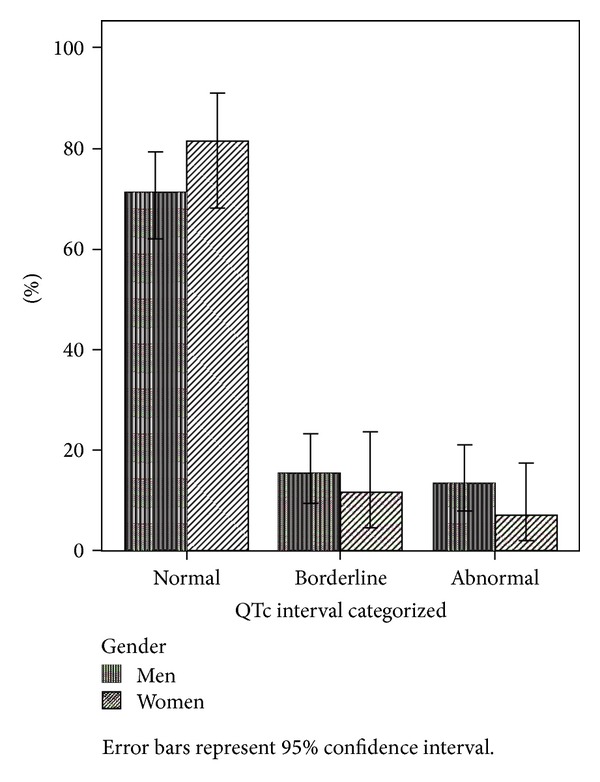
Distribution of QTc intervals at discharge/6 weeks.

A total of 95 patients were reassessed at discharge/6 weeks. Three of these patients were for practical reasons tested later than 6 weeks, at weeks 7, 10, and 11, respectively, but are included in the analyses. There were no significant differences between those tested only at baseline and those tested also at discharge/6 weeks with regard to baseline mean QTc interval or proportions with borderline or prolonged QTc intervals, neither were there baseline clinical or demographic differences. The mean QTc at discharge/6 weeks was 411.3 (23.1) ms. The proportions with prolonged or borderline QTc recordings were reduced to 4.2% and 5.3%, respectively (Figure 2). 

One patient had QTc interval > 500 ms and 4 patients had their QTc intervals increased more than 60 ms from baseline. The reduction of the proportion with prolonged QTc levels reached statistical significance (chi-square exact test: *P* = 0.046). There was a trend only for the reduction of the mean QTc (paired-samples *t*-test: *P* = 0.063; mean difference 7.5 ms; 95% CI −0.4–15.4). The use of antipsychotic drugs is displayed in Table 2. Two patients did not use antipsychotics at discharge/6 weeks.

The mean DDD with SD was 1.02 (0.55). There were no differences among the drug groups with respect to mean QTc interval or proportions with QTc prolongation. Concomitant antidepressants and/or mood stabilizers (lithium and anticonvulsants) and/or sporadic additional antipsychotics were used by 35.2%, 8.5%, and 18.3%, respectively, at discharge/6 weeks. There were no differences among the antipsychotic drug groups in this regard. There was no association between QTc level and DDD, or serum levels, respectively, of the antipsychotic drugs.

## 4. Discussion

The main findings of the present study in acutely admitted patients with psychosis were that almost a quarter of the patients had prolonged or borderline prolonged QTc intervals at hospital admission and this proportion was significantly reduced at discharge/6 weeks after the initiation of antipsychotic drug treatment with SGAs. Moreover, 2 patients at baseline and 1 patient at discharge/6 weeks had QTc intervals above the critical threshold of 500 ms, and 4 patients had their QTc intervals increased by more than 60 ms between baseline and discharge/6 weeks. The findings underscore that the acute admission situation may represent a particular risk phase with regard to QTc prolongation in this patient group. The baseline ECG recording was done before administration of the study drugs and although about half the sample had lifetime antipsychotic drug exposure it is reasonable to assume that only a minority had used their antipsychotics according to the prescription in the last period of time before admittance [28]. The high proportion with QTc prolongation at baseline must accordingly be explained also by factors other than the antipsychotic drugs. The reduced proportion with QTc prolongation at discharge/6 weeks further underscores this interpretation as one would expect that at least some of the SGAs under investigation should drive the QTc interval towards prolongation [2]. The results thus may suggest that in heterogeneous sample such as ours of acutely admitted psychosis patients the relative impacts on the QTc interval by antipsychotic drugs are outweighed by other factors including emotional stress.

Although the mean electrolyte levels were all within the reference ranges, there was a statistically significant negative association between serum calcium levels and the QTc intervals at baseline. Hypocalcemia has been shown to cause prolongation of the QTc interval [2, 4], and our results indicate that the association is present also within the reference range. Serum calcium levels in the lower end of the reference interval might accordingly represent an independent risk factor for QTc prolongation. By using the PANSS-EC as a proxy for emotional distress, we found a positive association between the agitation score and the QTc interval in the linear regression model. The study thus supports the findings indicated by Hatta et al. [18] that emotional distress may increase the QTc interval, contributing to the increased risk of QTc prolongation in active psychosis. Hatta et al. used the items anxiety, tension, mannerisms and posturing, hostility, uncooperativeness, and psychomotor excitement from the 18-item Brief Psychiatric Rating Scale as a collected agitation score and found that psychiatric emergency patients had had higher agitation scores compared to psychiatric outpatients and that the latter group had a significantly longer mean QTc interval. They did not however report analyses of direct comparisons between the QTc interval and agitation scores. Furthermore, we used the PANSS-EC as a proxy for emotional stress, which may not be directly comparable to the BPRS items chosen by Hatta et al.

Interestingly, the proportion with borderline or prolonged QTc intervals was significantly reduced from baseline to discharge/6 weeks and there were no differences among the drugs regarding QTc intervals at discharge/6 weeks which could indicate that the SGAs studied are safe in this regard. This interpretation of the data is strengthened by the fact that serum level measurements of the antipsychotics were conducted and, with the exception of aripiprazole which was used by only one patient, all the serum levels were in the middle of the respective reference intervals. Among the studied drugs ziprasidone was expected to have the longest QTc interval based on the previous literature [6]. Our finding of equality may suggest that drug differences found elsewhere are levelled out in more diagnostically heterogeneous samples such as ours. Alternatively, the present study may be underpowered resulting in a type II error but we find this unlikely given also the numerically equality among the drugs.

A major advantage of the present study is the consecutive inclusion of a diagnostically heterogeneous and thus clinically relevant sample with psychosis. The findings should accordingly be generalizable to patients acutely admitted to hospital for psychosis and eligible for oral antipsychotic medication. Based on this one could argue that ECG recordings should be mandatory in all psychosis patients acutely admitted to hospital. On the other hand the relationship between QTc prolongation and arrhythmias is not clear-cut [2] and may at best be considered a proxy for increased risk of malignant arrhythmias and sudden cardiac death. ECG is, however, an inexpensive investigation with minimal burden on the patient, and those patients at the highest risk of arrhythmia (QTc interval > 500 ms) can easily be detected.

Some potential limitations should be mentioned. Only three-quarters of the total sample underwent ECG recordings, thus reflecting that these measurements cannot be accepted by all acutely admitted patients with psychosis. One might expect that the most distressed patients were unable to cooperate with the procedure which was actually indicated by the higher PANSS-EC agitation score although not statistically significant and that these patients would have had even longer QTc intervals based on the positive association between agitation and the QTc interval. About half the sample had life-time exposure to antipsychotic drugs at study inclusion but noncompliance is a common problem in this patient group and a frequent cause of relapse [28], and most likely only a minority had used antipsychotic drugs according to their prescriptions in the last period of time before admittance. Serum drug levels were not measured at admittance, so the exact figures cannot be verified and accordingly comparisons between those taking and not taking antipsychotic drugs at baseline cannot be done although this would have added value to the study. There was a high attrition rate from baseline to discharge/6 weeks which could theoretically have introduced selection bias regarding the discharge/6 weeks recordings. We find this unlikely, however, as attrition was not related to any of the baseline variables. If present, the direction of any bias would be hard to predict. In our study the QTc interval was automatically recorded without manual control. The automated methods have however demonstrated noninferiority compared to manual recordings of the QTc but both methods have their flaws [29]. Finally, our study would have benefited from a control group for comparison. Little research is done on the QT interval in the normal population.

## 5. Conclusion

The results may indicate that ECG recordings should be mandatory in acutely admitted patients with psychosis to detect those at heightened risk of arrhythmias.

## Figures and Tables

**Table 1:** Baseline demographics and clinical characteristics.

Characteristics	*N*	% of sample
Gender		
Male	118	69.0
Female	53	31.0
Antipsychotic drug naïve	73	42.7
Alcohol use last 6 months		
None	33	19.3
Misuse	12	7.0
Illicit drug use last 6 months		
None	118	69.0
Misuse	29	17.0
Diagnosis^1^		
Schz and related	95	57.5
Acute	13	7.9
Drug-induced	21	12.7
Affective	19	11.5
Rest	17	10.2

	Mean	SD/range

Age	34.2	13.9/17–73
PANSS total	73.5	13.7/44–111
PANSS positive	19.7	4.4/11–32
PANSS negative	19.2	7.5/7–39
PANSS general	34.6	6.8/20–56
CDSS	6.9	5.3/0–23
GAF-F	30.9	6.0/10–62
CGI	5.2	0.6/4–6
RBANS, *t*-scores	38.3	7.5/20–58

*Notes*. *N* = number of patients with ECG at baseline and/or ECG at discharge; SD = standard deviation; antipsychotic drug naive = no life-time exposure to antipsychotic drugs before index admission; first admission = index admission was the first admission to a mental hospital; misuse = misuse or dependence according to the Clinical Drug and Alcohol Use Scales (CDUS/CAUS); patients with no illicit drug use could be included in the category alcohol use last 6 months; Schz and related = schizophrenia and related disorders: schizophrenia, schizoaffective disorder, acute polymorphic psychotic disorder with symptoms of schizophrenia, acute schizophrenia-like psychotic disorder, and delusional disorder; acute = acute psychosis other than those categorized under Schz and related; affective = affective psychosis; rest = miscellaneous psychotic disorders. All diagnoses are according to ICD-10; PANSS = the positive and negative syndrome scale; CDSS = the Calgary Depression Scale for Schizophrenia; GAF-F = the Global Assessment of Functioning—Split Version, Functions scale; CGI = the Clinical Global Impression, Severity of Illness scale; RBANS = the repeatable battery for the assessment of neuropsychological status.

^1^Patients with missing diagnoses are not included in the list.

**Table 2:** Antipsychotic drug use and QTc intervals at discharge/6 weeks.

	Risperidone *N* = 25	Olanzapine *N* = 31	Quetiapine *N* = 18	Ziprasidone *N* = 18	Aripiprazole *N* = 1
	Mean (SD)	Mean (SD)	Mean (SD)	Mean (SD)	Mean (SD)
Mean dose (mg/d)	3.5 (1.2)	16.4 (5.9)	457.4 (222.2)	86.7 (39.0)	5.0 (—)
Serum level (nm/L)*	71.6 (47.9)	121.6 (77.7)	501.9 (555.1)	125.3 (99.8)	141 (—)
QTc (ms)	411.3 (24.6)	411.6 (27.0)	412.6 (16.4)	412.5 (19.9)	362.0 (—)

*Notes*. *N* = number of patients; SD = standard deviation; mg/d = milligrams per day; nm/L = nanomoles per litre; ms = milliseconds.

*Reference ranges: risperidone 30–120; olanzapine 30–200; quetiapine 100–800; ziprasidone 30–200; aripiprazole 200–1300.
